# An *Ascophyllum nodosum*-Derived Biostimulant Protects Model and Crop Plants from Oxidative Stress

**DOI:** 10.3390/metabo11010024

**Published:** 2020-12-31

**Authors:** Nikola S. Staykov, Mihail Angelov, Veselin Petrov, Pavel Minkov, Aakansha Kanojia, Kieran J. Guinan, Saleh Alseekh, Alisdair R. Fernie, Neerakkal Sujeeth, Tsanko S. Gechev

**Affiliations:** 1 Center of Plant Systems Biology and Biotechnology, 4000 Plovdiv, Bulgaria; angelov@cpsbb.eu (M.A.); petrov@cpsbb.eu (V.P.); kanojia@cpsbb.eu (A.K.); alseekh@mpimp-golm.mpg.de (S.A.); Fernie@mpimp-golm.mpg.de (A.R.F.); gechev@cpsbb.eu (T.S.G.); 2 Department of Plant Physiology, Biochemistry and Genetics, Agricultural University, 4000 Plovdiv, Bulgaria; 3 Institute of Molecular Biology and Biotechnology, 4000 Plovdiv, Bulgaria; pvl.minkov@gmail.com; 4 BioAtlantis Ltd., Tralee, V92 RWV5 Co. Kerry, Ireland; research@bioatlantis.com (K.J.G.); Plant.Research@BioAtlantis.com (N.S.); 5 Max Planck Institute of Molecular Plant Physiology, 14476 Potsdam, Germany; 6 Department of Plant Physiology and Molecular Biology, Plovdiv University “Paisii Hilendarski”, 4000 Plovdiv, Bulgaria

**Keywords:** *Ascophyllum nodosum*, biostimulant, oxidative stress

## Abstract

Abiotic stresses, which at the molecular level leads to oxidative damage, are major determinants of crop yield loss worldwide. Therefore, considerable efforts are directed towards developing strategies for their limitation and mitigation. Here the superoxide-inducing agent paraquat (PQ) was used to generate oxidative stress in the model species *Arabidopsis thaliana* and the crops tomato and pepper. Pre-treatment with the biostimulant SuperFifty (SF) effectively and universally suppressed PQ-induced leaf lesions, H_2_O_2_ build up, cell destruction and photosynthesis inhibition. To further investigate the stress responses and SF-induced protection at the molecular level, we investigated the metabolites by GC-MS metabolomics. PQ induced specific metabolic changes such as accumulation of free amino acids (AA) and stress metabolites. These changes were fully prevented by the SF pre-treatment. Moreover, the metabolic changes of the specific groups were tightly correlating with their phenotypic characteristics. Overall, this study presents physiological and metabolomics data which shows that SF protects against oxidative stress in all three plant species.

## 1. Introduction

Abiotic stress, provoked by extreme temperatures (heat or cold), water shortage or excess caused by drought or flooding, high light and UV radiation, salinity, heavy metals and other pollutants, or their combinations, has been recognized as a major source of economic damage to agriculture [1,2]. A common feature of all these events is that they lead to the accumulation of the so-called reactive oxygen species (ROS) in plants, including hydrogen peroxide—H_2_O_2_; superoxide—O_2_^•−^; hydroxyl radical—HO^∙^, singlet oxygen—^1^O_2_, etc. [3,4]. In low concentrations ROS exert signaling functions and may prime the plants for enhanced stress tolerance[5]. However, due to their high oxidizing potential, at high doses they can damage numerous cell constituents and even trigger programmed cell death (PCD) [6,7,8]. Treatment of plants with ROS-inducing agents like aminotriazole, menadione and paraquat (PQ), or directly with H_2_O_2_, is a widespread approach in studies related to abiotic stress since it allows the easy and reliable identification of sensitive or tolerant individuals in different experimental setups [9].

In recent years, an elegant solution to optimize plant growth and performance, which attracts the attention of farmers, the industry and the research community, is the treatment with metabolic enhancers called biostimulants [10,11,12]. According to the European Biostimulant Industry Council (EBIC) these products “contain substance(s) and/or microorganisms whose function when applied to plants or the rhizosphere is to stimulate natural processes to enhance/benefit nutrient uptake, nutrient efficiency, tolerance to abiotic stress, and crop quality”. Biostimulants are reported to improve yield both in normal conditions and under slight to moderate stress [11]. Moreover, they are also regarded as an alternative to traditional agro-chemical practices with the potential to reduce the usage of pesticides and fertilizers [13]. Biostimulants are obtained from natural sources and have undefined chemical composition based on a mixture of multiple chemicals like vitamins, minerals, carbohydrates, amino acids and peptides, phenolic compounds, etc. [11,14]. Due to their complexity, it is possible that the observed beneficial properties of these products may be caused by the synergistic action of their different components rather than by the properties of individual constituents. Depending on their source, biostimulants can be divided into different categories, the most prominent of which are prepared from: microorganism isolates, protein hydrolysates of animal or plant origin, seaweed extracts, humic/fulvic acids, etc. [14].

Among these different categories, one of the most successful on the market includes the products harvested from seaweeds [15]. This is a very diverse group of biostimulants whose exact properties depend on the source species, the methods used for extraction, the conditions in the areas used for harvesting, etc. [14,16]. Some of the reported beneficial effects of seaweed extracts on plant vigor involve improvement of the root development, synchronization of the fruits, acceleration of fruit onset and flowering, delayed senescence and enhanced stress tolerance [17,18,19]. Another important aspect that contributes to the popularity of these products is their easy biodegradability and lack of toxicity to the environment and human health [20]. Crops, for which positive influence of seaweed extracts on yield and quality has been described in the literature, include wheat, rice, tomato, cucumber, broccoli, spinach, bean, etc. [14,21].

Biostimulants produced from the brown inter-tidal alga species *Ascophyllum nodosum* (L.) of the *Fucaceae* family not only have significant commercial success but are also widely studied [12,22]. As a result, several modes of action of *Ascophyllum nodosum* extracts (ANE) in eliciting plant stress mitigation was recently proposed [12]. A specific ANE product available on the market, called SuperFifty (SF), has demonstrated high potential as a potent promoter of plant growth and productivity [18,23] mainly by reducing stress and enhancing fruit setting processes in plants. SF has been shown to ameliorate plant water holding capacity via modulating the ABA-dependent pathway and partial closure of stomata (unpublished data). However, details on the protective properties of SF on different economically important crops as well as its precise mechanism of action are necessary in order to fully characterize this product. Since the effects of a given biostimulant may differ among different species [11], in the current work two important crops—tomato (*Solanum lycopersicum* cv. MoneyMaker) and sweet pepper (*Capsicum annuum* cv. Kurtovska Kapiya-1), as well as the model plant *A. thaliana* (ecotype Col-0) were studied. Their physiological and biochemical responses to oxidative stress provoked by PQ and the ability of SF to alleviate the resulting stress symptoms were compared. Details on the underlying molecular mechanisms were obtained by monitoring the primary metabolite profiles and their rearrangements after treatments with SF and/or PQ in all three species.

## 2. Results

Oxidative stress in the tissues of Arabidopsis, tomato and sweet pepper was induced by spraying plants at the developmental stage of two fully grown leaf pairs (for pepper and tomato) and the stage of rosette just before bolting (for Arabidopsis) with the herbicide paraquat. This chemical causes superoxide-dependent cell death by accumulation of excess amounts of superoxide anions. The PQ-application was repeated a second time 24 h later. Half of the PQ-treated plants were preliminarily pre-treated with SF-solution by spraying their leaves one day before applying the stressor. The goal of this pre-treatment was to test the ability of SF to protect the living tissues from the adverse effects of the following ROS-induction. Application concentrations for PQ and SF were pre-determined in preliminary experiments (for SF see Supplementary Figure S1).

In addition, two controls were used—non-treated negative control and SF-treated only, creating a total of four studied variants: −SF/−PQ (not treated); +SF/−PQ (SF-only treated); −SF/+PQ (PQ-stressed); +SF/+PQ (SF pre-treated and PQ-stressed). Several physiological and biochemical properties of the tested specimens were examined.

### 2.1. Pre-Treatment of Plant Leaves by Spraying with SF-Solution Strongly Protects from PQ-Induced Damage

**Figure 1:**
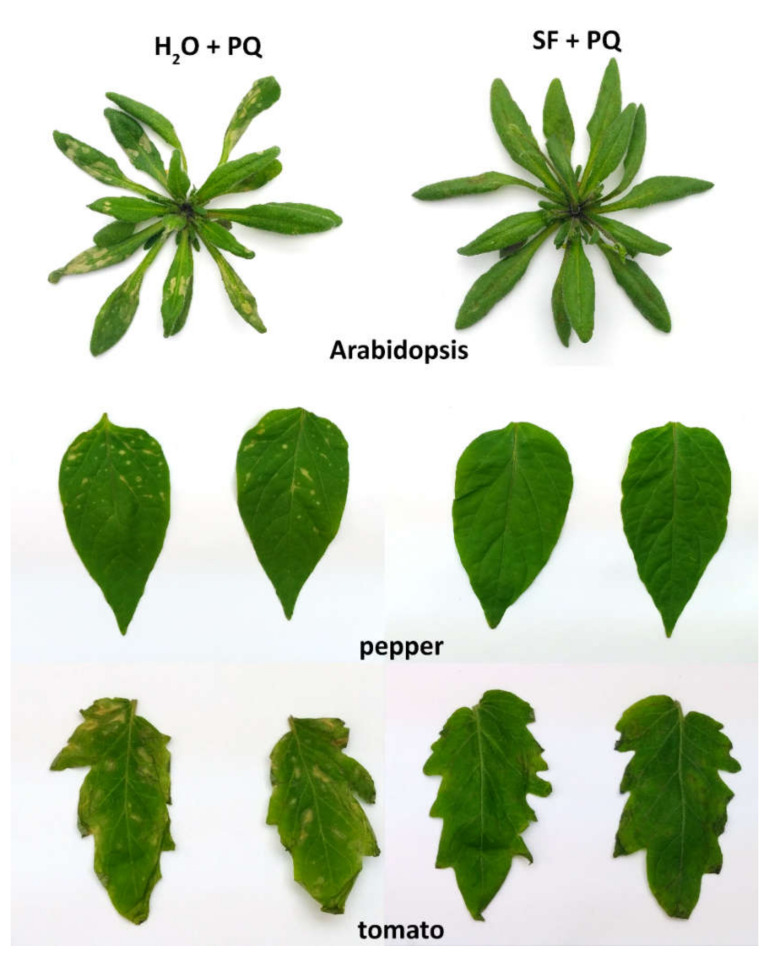
SuperFifty (SF) protects leaves from damage, caused by oxidative stress. Plants on the left are treated with paraquat (PQ), a herbicide that elevates the endogenous levels of ROS and triggers cell death (15 µM for Arabidopsis and 25 µM for tomato and pepper), and have clearly visible lesions. Plants on the right were pre-treated with 1% aqueous solution of SF, followed by the same treatment with PQ. Control plants, sprayed only with water (H_2_O) or only with 1% SF, show no signs of effect (pictures not shown).

The visible damage on the leaves, following PQ-treatments, appeared in the form of rounded lesions on the parenchyma. This was the resulting observable phenotype, caused by the process of programmed cell death, induced by the elevated amounts of ROS [24]. Since dissimilar species are differentially sensitive to this process, it was necessary to adjust the PQ concentrations according to the individual tolerance levels in order to achieve comparable phenotypes: 25 µM for tomato and pepper and 15µM for Arabidopsis. Whole leaves and rosettes were photographed 24 h after the second PQ application. As it is seen on the pictures below (Figure 1), pre-treating with SF before spraying with PQ causes a clear protective effect on the three studied species. SF-sprayed plants show almost no damage after subsequent PQ application, whereas those that were not pre-treated with the biostimulant developed substantial lesions.

### 2.2. The Total Size of All Developed Lesions Drops Close to Base Levels, When a Single Pre-Treatment with SF Is Applied

**Figure 2:**
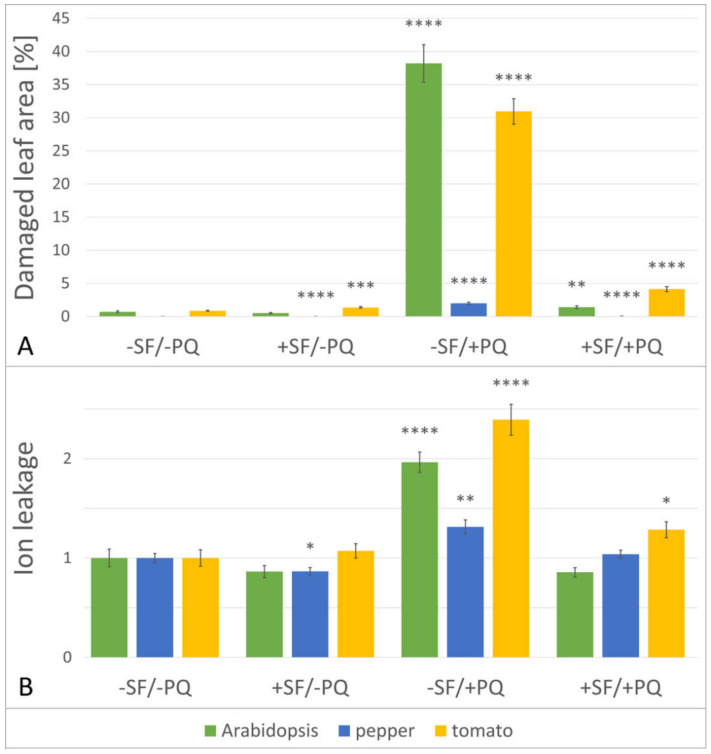
Damage reduction, caused by SF pre-treatment. (**A**) SF reduces leaf lesion area in PQ-treated plants. The dead leaf surface, as a parameter indicating cell damage, of the four variants (as described in Materials and Methods) of Arabidopsis, pepper, and tomato (green, blue and yellow color bars, respectively) was assessed with the ImageJ software. *n* = 12 for Arabidopsis, *n* = 48 for pepper and tomato. (**B**) SF prevents excess ion leakage in PQ-treated plants. Measurement of electrolyte leakage in leaves of Arabidopsis, pepper and tomato exposed to four different treatments, relative to (−SF/−PQ) control. *n* = 36 for Arabidopsis, *n* = 48 for pepper and tomato. Data are means ±SEM (standard error of the mean) of three biological replicates. Differences between two single treatments were tested by Welch’s t-test, with *p* < 0.05. Statistical significance relative to controls from the same species is indicated with asterisks as follows: *p* ≤ 0.0001 (****), *p* ≤ 0.001 (***), *p* ≤ 0.01 (**), *p* ≤ 0.05 (*).

To obtain a quantitative descriptor of the observed oxidative stress mitigation by the biostimulant, we compared the total rate of lesion formation under the four different conditions. Using the individual photos of the plant phenotype, the cumulative area of all lesions was calculated and plotted as a percentage ratio of the total leaf area (Figure 2, panel A). After SF-pretreatment, a very strong decrease of the PQ-damaged areas for all species was observed, suggesting that the processes leading to cell death were significantly alleviated. Even though on the composite graph the total lesion areas of pepper have the smallest absolute values, when the stressed samples (−SF/+PQ) are compared to the SF-rescued group (+SF/+PQ), it turns out that pepper actually shows the highest rescue effect of 37-fold decrease of lesion area, followed by Arabidopsis with 27-fold and tomato with 7-fold decrease.

### 2.3. Pre-Treatment with SF Decreases the Membrane Damage during PQ-Induced Oxidative Stress

Electrolytes contained within the cell, leak out when plants are exposed to severe stress conditions or during the onset of senescence, as the integrity of the cell membranes is compromised [25]. Measuring the leaked solutes from plant tissues is a very common physiological method to determine the intactness of cell membranes [26], which is a marker of stress intensity. Electrolyte leakage (EL) relative to control (−SF/−PQ) was calculated for Arabidopsis, pepper and tomato leaves treated or not with SuperFifty and/or paraquat.

The results show that ion leakage is invariant between −SF/−PQ and +SF/−PQ samples in Arabidopsis and tomato, while in pepper there was a slight reduction after application of SF (Figure 2, panel B). By contrast, as expected the percentage of leaked ions significantly increased in PQ only treated samples (−SF/+PQ) in all three species. Remarkably, the pre-treatment with SF of PQ stressed leaves (+SF/+PQ) resulted in considerably reduced amounts of ion leakage, demonstrating the protective properties of SF and the effective mitigation of the applied oxidative stress. In the case of Arabidopsis and pepper, the EL values returned to the control levels, while in tomato they remained slightly higher.

### 2.4. SF Application Strongly Reduces the Hydrogen Peroxide Accumulation in the Leaf Tissues

The mode of action of paraquat stress goes through accumulation of extra amounts of superoxide anions, which are short-lived and in turn can be transformed to the more stable H_2_O_2_ molecule [27]. The latter persists longer in the cells and is easily detectable by histochemical staining with 3, 3′-diaminobenzidine (DAB) [28]. To investigate whether the protective action of SF includes suppression of ROS generation, the accumulation of H_2_O_2_ was monitored in Arabidopsis, pepper and tomato leaves by treatment with DAB solution.

**Figure 3:**
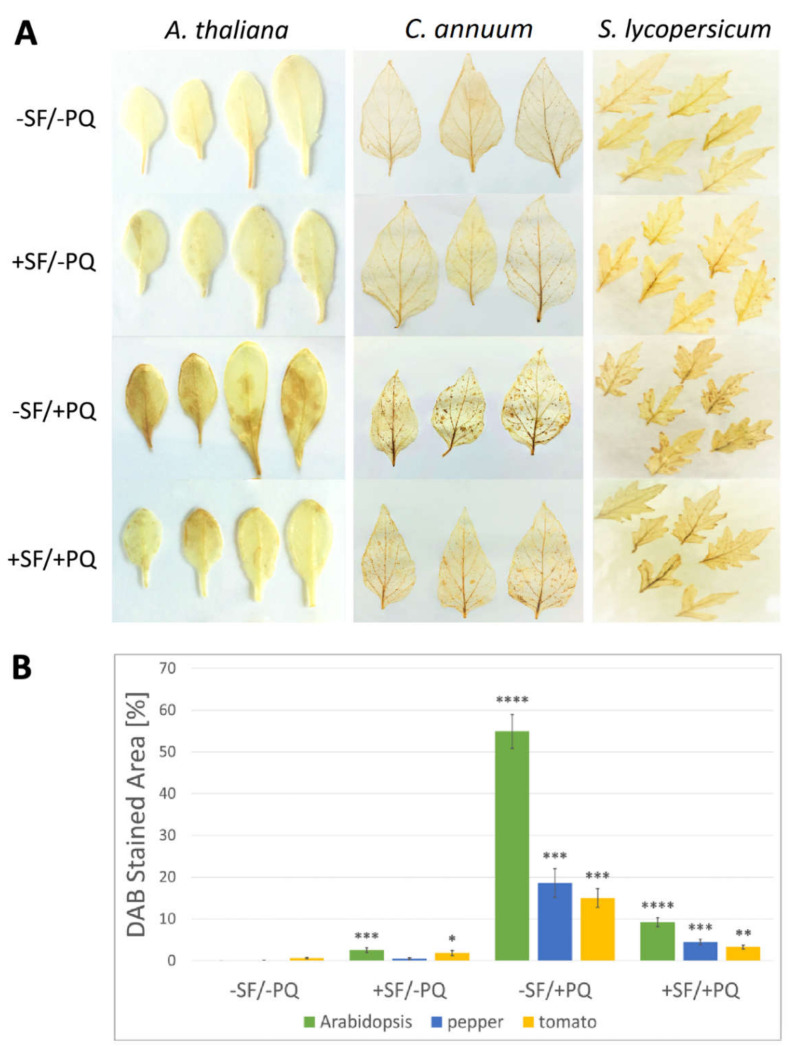
SF protects from accumulation of H_2_O_2_ in PQ treated plants. (**A**) DAB-stained leaves of Arabidopsis, pepper and tomato exposed to different treatments (**B**) The percentage of DAB-stained area of the four variants of Arabidopsis, tomato, and pepper was assessed with the ImageJ software as described in Materials and Methods (*n* = 6). Data are means ±SEM of three biological replicates. Differences between two single treatments were tested by Welch’s t-test, with *p* < 0.05. Statistical significance relative to controls from the same species is indicated with asterisks as follows: *p* ≤ 0.0001 (****), *p* ≤ 0.001 (***), *p* ≤ 0.01 (**), *p* ≤ 0.05 (*).

The level of stained H_2_O_2_ appears to be low in both −SF/−PQ and +SF/−PQ leaves, as no apparent brown precipitation was observed (Figure 3, panel A). On the other hand, in PQ treated leaves (−SF/+PQ) the stained H_2_O_2_ is more prominent in all the three species. The pre-treatment with SF of PQ-stressed leaves clearly leads to an overall reduced area of brown spots, confirming that SF is able to hinder ROS accumulation in stressed tissues. The graph of Figure 3 (panel B), shows the percentage of the area of DAB-stained lesions under the different conditions. In PQ-only treated Arabidopsis, pepper and tomato leaves, 55%, 18% and 15% of the leaf area, respectively, was stained. By contrast, +SF/+PQ samples displayed only 9%, 4% and 3% stained area in Arabidopsis, pepper and tomato, respectively. These results suggest that the mechanism through which SF protects the living tissues includes limitation of the oxidative load. It must be noted that the effect of PQ does not seem to be completely blocked by SF, since some H_2_O_2_ buildup is detected also in the +SF/+PQ variants.

### 2.5. PAM-Fluorometry Measurements Confirm the Rescue Effect of Pre-treatment with SF

**Figure 4:**
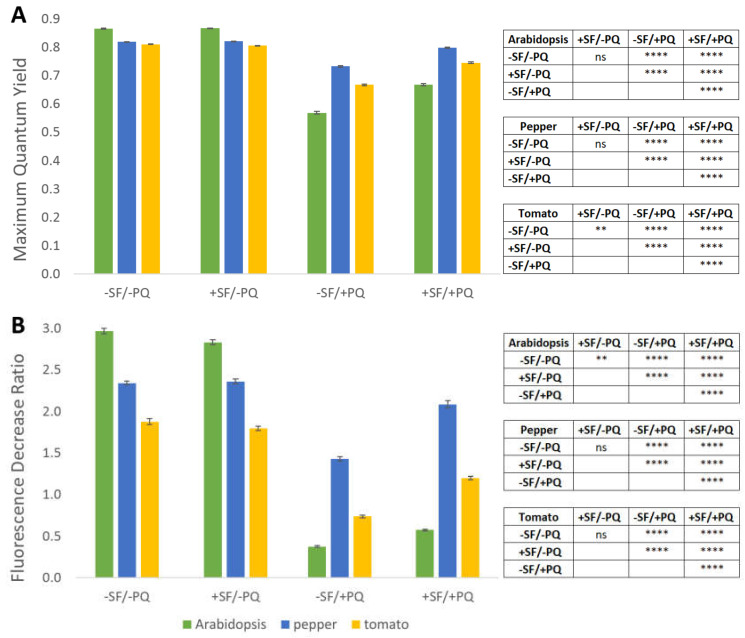
SF pretreatment alleviates the reduction of chlorophyll fluorescence parameters induced by oxidative stress. The maximum quantum yield (**A**) and fluorescence decrease ratio (**B**) in dark-adapted Arabidopsis, pepper and tomato plants (*n* = 12), treated or not with SF and PQ, were measured by PAM fluorometry. Data are means ±SEM of three biological replicates. Differences between two single treatments were tested by Welch’s t-test, with *p* < 0.05. Statistical significance relative to controls from the same species is indicated in the Table as follows: *p* ≤ 0.0001 (****), *p* ≤ 0.001 (***), *p* ≤ 0.01 (**), ns = not significant.

Photosynthesis is one of the most sensitive physiological processes in plants and it is easily disturbed by stress conditions [29]. To observe the status of the photosynthetic apparatus (PSII) in the investigated experimental variants, chlorophyll fluorescence measurements were carried out on dark adapted plants with a Pulse Amplitude Modulation (PAM) fluorometer. The maximum quantum yield (QY_max_) and fluorescence decrease ratio (R_Fd_) parameters, both directly dependent on the oxidative stress levels and related damage on the photosystems, are presented on the next graph (Figure 4). The QY_max_ reflects the portion of light that is used in photochemical reactions, while the R_Fd_ coefficient is linearly correlated to the CO_2_ fixation rate [30].

As shown in Figure 4, the findings from this experiment support the earlier observations on the plant phenotype, stress load and damaged areas. The application of SF alone does not have noticeable impact on both quantified photosynthesis-related parameters. As expected, treatment with PQ leads to a considerable reduction of QY_max_ and R_Fd_, with the effect on R_Fd_ being much more pronounced. In accordance with the previous data, spraying the plants with SF before the stress event significantly alleviates the negative effect of PQ, although both QY_max_ and R_Fd_ cannot reach the values measured in the controls. Of the three studied species Arabidopsis appears to have the most sensitive photosynthetic apparatus with respect to PQ since QY_max_ and R_Fd_ values change the most drastically in response to the stressor, decreasing by about 34% and 87%, respectively. The variation of the same parameters in pepper considerably lesser, being around 11% and 39%, respectively. Tomato plants hold an intermediate position with close to 18% and 61%, respectively. The rescue effect of the same parameters is, however, not as evenly distributed among the plant species. Although QY_max_ follows exactly the same pattern with 14.8%/8.3%/10.5% recovery for Arabidopsis/pepper/tomato, the R_Fd_ slightly differs from it, with the strongest rescue effect for tomato (38.5%), followed by Arabidopsis (34.4%) and pepper (31.5%).

### 2.6. Application of PQ and SF Cause the Formation of Distinct Patterns of Metabolite Abundance 

To shed light on the underlying molecular mechanisms involved in the occurrence of the rescue effect achieved by supplementation with SF, the primary metabolome and its reconfigurations imposed by the different treatments were investigated by GC-MS.

We monitored the changes in abundance of 89 metabolites for each of the three studied species and the four treatment variants. Principal Component Analysis (PCA) of double-normalized datasets (as described in Materials and Methods), calculated from the total amount of metabolites, was carried out using MetaboAnalyst and 2D PCA Scores plots were generated. PCA is an unsupervised pattern recognition method, aiming to determine the degree to which all variables in the data set are related [31]. We used this technique to identify potential tendencies for each particular treatment to form specific groups of metabolite distribution. As a result, for all three plant species, the majority of the treatments displayed partially or completely overlapping patterns, with the exception of −SF/+PQ, which clustered mostly separately, overlapping only with the 95%-confidence regions (see Supplementary Figure S2). In pepper though, the −SF/+PQ treatment concurred with the other groups more than in the other plants, suggesting a weaker effect of PQ on the metabolome. Also in pepper, the +SF/−PQ treatment displayed a much larger variation between samples, forming very wide 95%-confidence region.

**Figure 5:**
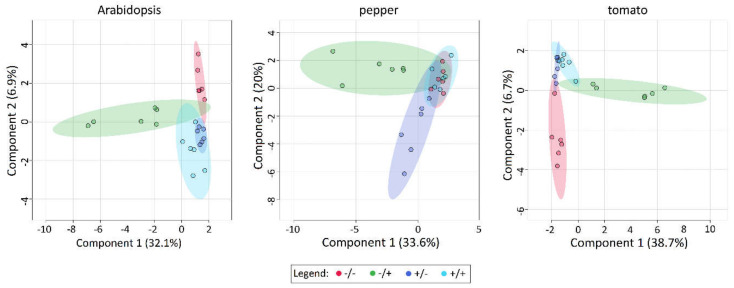
2D sPLSDA Scores plots for 89 metabolites of Arabidopsis, pepper and tomato. The −SF/+PQ treatment (light green horizontally arranged clusters) always groups separately from the others, which showing much more similar distribution and partial or complete overlapping (all others, generally vertical clusters) (*n* = 6). AT—*Arabidopsis thaliana*, CA—*Capsicum annuum*, SL—*Solanum lycopersicum*.

In contrast to PCA, Sparse Partial Least Squares Discriminant Analysis (sPLSDA) is a supervised technique, where each sample’s group is defined in advance, enabling more efficient separation [32]. The same datasets were additionally analyzed with the sPLSDA function of MetaboAnalyst and the 2D Scores plots were compared. The sPLSDA analysis demonstrates that a high level of variation in the metabolome of Arabidopsis, pepper and tomato among treatment conditions can be explained by the first component (>32.1%, 33.2% and 38.5% respectively; Figure 5). In each of the sPLSDA plots, the oxidatively stressed group (−SF/+PQ) deviates from the other treatment groups, an effect observed for each plant species assessed. In contrast, the remaining treatments (−SF/−PQ, +SF/−PQ and +SF/+PQ), clustered closely together along the first component. This indicates a high similarity at the metabolomic level between the SF-treated plants and unstressed plants, which closely resembles the observations from the phenotype analysis (Figure 2, Figure 3 and Figure 4). Comparably to the PCA plot (Supplementary Figure S2), in the pepper sPLSDA, +SF/−PQ had a high variation, although clearly divided from the rest of the treatments. In this crop, +SF/+PQ completely overlapped with the negative control, indicating high degree of rescue, while in the other two species, the overlapping occurred between +SF/−PQ and +SF/+PQ variants.

Furthermore, examination of Synchronized 3D sPLSDA plots revealed that the formerly overlapping 2D groups diverge in three separate aggregations not sharing any points of contact in the three-dimensional space, when the third axis of component 3 is added to the graph (see Supplementary Figure S3). This indicates the existence of subtle differences in their metabolic profiles, probably due to different responses to the treatment.

**Figure 6:**
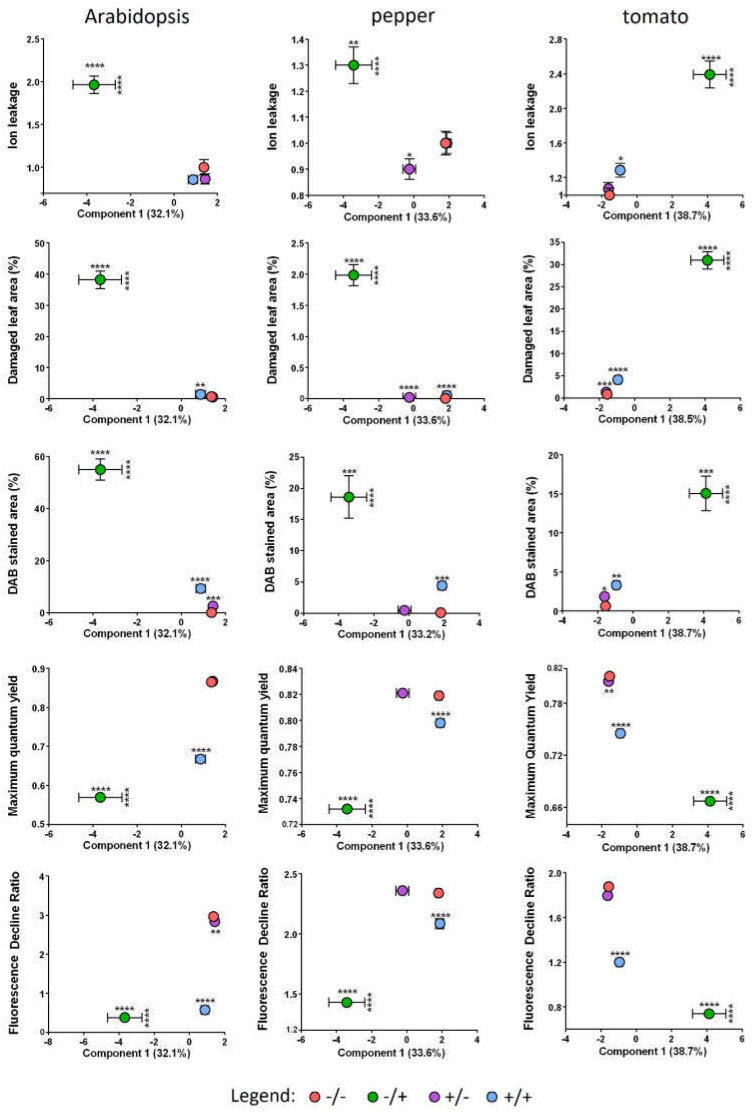
Scatter plots of plant phenotype parameters versus sPLSDA component 1 values. Mean sPLSDA component 1 values were compared between treatment groups using one-way ANOVA, with Tukey’s multiple comparisons test applied to correct for multiple testing. For all three plant species, a significant difference was observed between the −SF/+PQ (oxidatively stressed) and all other treatment groups. This was observed for both the sPLSDA component 1 values and for all plant phenotypes measured. Horizontal error bars denote the standard error of the mean (SEM) of sPLSDA component 1 values. Vertical error bars denote the SEM of phenotype parameters. Asterisks denote statistically significant differences between treatment groups and the untreated control (−SF/−PQ) as follows: *p* ≤ 0.0001 (****), *p* ≤ 0.001 (***), *p* ≤ 0.01 (**), *p* ≤ 0.05 (*). Horizontal and vertical asterisks denote significant differences between treatment groups and the untreated control for phenotype parameters and sPLSDA component 1 values respectively. Full data are given in Supplementary Materials (Supplementary Figures S4 and S5) and Supplementary Table S1.

A scatter plot of plant phenotypes and sPLSDA component 1 values demonstrates a close clustering of the SF treated plants with unstressed plants (Figure 6). For all three species, the +SF/+PQ group was observed to cluster close to the unstressed groups (+SF/−PQ and −SF/−PQ), particularly in the case of ion leakage, damaged leaf area and DAB staining. In terms of the maximum quantum yield and fluorescence decline ratio, a similar distribution was observed in pepper, with +SF/+PQ clustering closely with the unstressed groups (+SF/−PQ and −SF/−PQ). In the case of Arabidopsis and tomato, the +SF/+PQ group clustered between the unstressed groups (+SF/−PQ and −SF/−PQ) and the oxidatively stressed group (−SF/+PQ). Close clustering according to phenotype was not observed for the second component which accounts for less of the variation in metabolic changes between groups (Figure 5 and Supplementary Figure S4). Overall, these data indicate that the metabolomic changes induced by SF are strongly correlated to the visible plant phenotypes obtained. Moreover, the data suggest that treatment with SF prevents stress responses being induced by PQ. This effect is manifested at both the metabolomic and phenotypic level and for all three plant species assessed.

To investigate the metabolite configurations, characteristic for each of the treatments for each of the plant species, clustered heat maps of metabolite amounts, relative to the corresponding negative control (−SF/−PQ) were generated (Supplementary Figure S6). A general tendency of the −SF/+PQ samples to contain metabolites arranged in a different pattern than the other variants was detected among all species.

In each species, a very small number of metabolites has characteristically changed amounts in SF- treatments only. They can be considered as part of the specific response to SF. Only one case of accumulation of a metabolite was observed in Arabidopsis (unknown_sugarSuper50), while for each of pepper and tomato they are three (4-hydroxy-proline, guanidine, maltose and 2-methyl-malic acid, maltose and glycerol-2-phosphate, respectively). In the latter two species, there is also one example of SF-induced down-accumulation of a compound (quinic acid in both).

**Figure 7:**
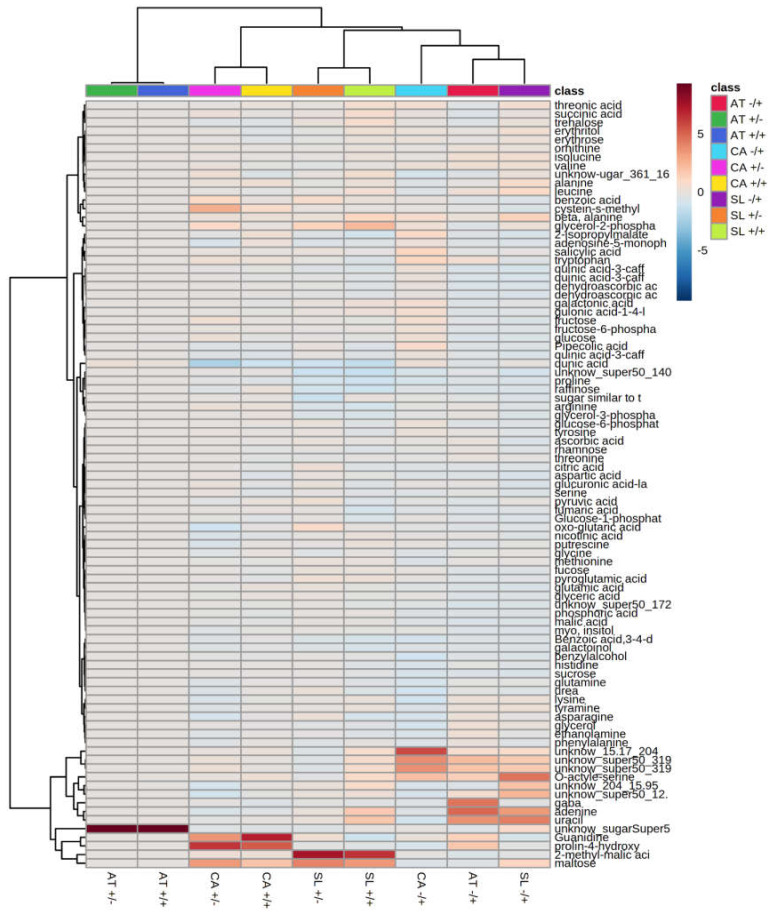
Levels of primary metabolites, relative to the negative control (−SF/−PQ) for each of the species, clustered for similarity of distribution patterns. The length of the lateral arms of the dendrograms represent the degree of resemblance. Significance can be seen in Supplementary Table S2 (*n* = 6). AT—*Arabidopsis thaliana*, CA—*Capsicum annuum*, SL—*Solanum lycopersicum*.

Assessing the inter-species degree of similarity of the metabolite patterns was done by clustering together all of the independent treatments (after normalization to the respective −SF/−PQ controls; Figure 7). Intriguingly, all PQ-treated variants grouped apart, leaving the other two treatments from each species closely paired, with greater similarity between the Arabidopsis couple than the *Solanaceae* ones. This means that the Arabidopsis groups +SF/−PQ and +SF/+PQ are the most similar to each-other and to the respective −SF/−PQ control than any of the other two pairs.

Three out of the top 25 modulated metabolites in the −SF/+PQ variants displayed similar distribution patterns (either up or down accumulation), clearly differing from the other treatments for the same species (Supplementary Figure S7). One of them is down-accumulated (2-methyl-malic acid) and the two up-accumulated are unidentified substances which may be of interest for further investigation. In addition to these commonly changed metabolites, in several instances specific substances are modulated in some plant species only, but not in others. Examples include “unknown_15.17_204”, accumulated only in −SF/+PQ pepper and “unknown_204_15.95” plus “unknown_super50_12.17_361_337”, accumulated only in −SF/+PQ tomato (Figure 7).

With the aim of identifying only the stress-responsive compounds that were affected by the SF pre-treatment, we further restricted the selection to only those, answering all of the three following conditions at once: −SF/+PQ treatment to be significantly different vs. −SF/−PQ (indicative of a stress-dependent response); +SF/−PQ not to be significant vs. −SF/−PQ (not influenced by SF application alone); and +SF/+PQ to be significant vs. −SF/+PQ (demonstrating a rescue effect resulting from SF-pretreatment). The list of metabolites covering these criteria decreased to 35 for Arabidopsis, 32 for tomato and only six for pepper (Supplementary Figure S8). For Arabidopsis, nine were down-accumulated in the variant −SF/+PQ and the rest up-accumulated; for tomato—two down-accumulated and the rest up-accumulated; and for pepper—all were up-accumulated. Though the abundance of these compounds in the +SF/+PQ variant does not always return to the control amounts (−SF/−PQ) and in some occasions is still considerably different from them (for example uracil in Arabidopsis), the rescue effect is always very pronounced and significant compared to −SF/+PQ. In these cases, it can be considered partial rescue.

Considering the effect of the combined treatment (+SF/+PQ), for Arabidopsis 10 metabolites were down-accumulated and 25 up-accumulated compared to the control (Supplementary Figure S8). Interestingly, for five of these the effect of SF was the opposite to the one of PQ. For tomato—10 compounds were down-accumulated and 22 up-accumulated, with eight showing contrasting responses to SF and PQ. Finally, for pepper four out of six molecules were depleted in +SF/+PQ, while in the stressed −SF/+PQ variant their levels increased.

## 3. Discussion

SuperFifty^®^ (SF) is a commercially available biostimulant which contains highly concentrated A. nodosum extract (500 g/L) [33]. It is a biodegradable product, highly rich in functional bioactives and antioxidant substances such as phlorotannins and fucoidans [12], as well as proline (involved in drought and salt stress tolerance), mannitol (osmoprotectant and ROS scavenger protecting photosynthesis during abiotic stress) and sorbitol (free radical scavenger) [18]. The stress reducing potential of SF is possibly due to the presence of uncommon or unique polysaccharides such as fucoidan [19]. These polysaccharide molecules may prime and trigger plant signaling pathways [19,34].

Due to these properties, SF is proven to enhance abiotic stress tolerance in various plants and helps to improve fruit quality, crop yield, root growth and plant growth [12,23,33,35]. The polyphenol content and antioxidant capacity were found to be much higher in SF than in the similar product Ecolicitor^®^ [33]. ANE extracts also induce nutrient uptake in plants and it was shown that SF application significantly enhanced the accumulation of antioxidants, minerals and essential amino acids in tomato fruits [18]. The effects of SF and other biostimulants on the metabolome are also increasingly being assessed ([19,36] and references therein).

The antioxidant characteristics of SF and several of its components make it a suitable candidate for investigating its relationship with an oxidative stress simulation system. Indeed, a recent study demonstrated that SuperFifty foliar application rescued paraquat-induced oxidative damage in Arabidopsis plants [19]. To our knowledge, this is the first comparative survey simultaneously carried out with three species, which demonstrates the similarities and differences in their physiological and biochemical responses after individual and combined application of a ROS-inducer and the biostimulant. As the aim of this work was not to study influences on the yield, seed production, germination or any other agricultural properties of the plants, but rather to examine some of their faster reactions, the vegetative phase of their development which was selected for investigation, had to serve several purposes: to allow comparison with previous studies, to be a relatively early stage of development, and to ensure convenient plant material management. Considering these requirements and the interest in the short-term responses, a scheme with pre-treatment (preventive action) with SF was chosen, rather than post-treatment (healing effect). The rationale is to be able to prevent the detrimental effects of the stress rather than repair the already received damages, which is preferable from a practical point of view.

The three investigated plant species manifested specific reactions to the same stressor and displayed dissimilar amounts and type of damages, including shape, pattern, distribution and coloring of the affected areas. Arabidopsis formed the largest lesions, widely distributed among the whole leaf area, while pepper leaves developed only small round lesions, grouped mostly on the basal half of the leaf blades. Tomato had an intermediate position in this aspect (Figure 1). Based on these observations and considering the lower concentration of PQ in the Arabidopsis treatments, we can suggest that under these conditions, Arabidopsis shows the highest sensitivity to the stress and pepper the lowest.

Pre-treatment with SF produced a well-marked rescue effect, with different strength for the three species. The degree of protection was the strongest for the least sensitive species - pepper, slightly weaker for the most damaged Arabidopsis and the weakest, but still significant, for tomato (Figure 2). The reasons for the pronounced individual responses to the stressor and the biostimulant may be related to the differences in species anatomy and/or physiology, such as thinner or thicker cuticle, absence or presence of trichomes, number and size of stomata on treated surfaces, etc. Another plausible explanation is the potentially different mechanisms of action, through which SF promotes its protective effect.

The DAB-staining experiment showed regions of H_2_O_2_ accumulation with a very similar pattern, size and shape to the visible lesions. The high degree of their overlap supports that the lesions are caused by locally forming ROS bursts. SF pre-treatment causes a noticeable decrease of the H_2_O_2_-stained areas, suggesting that its mechanism of action includes prevention of ROS buildup (Figure 3).

Additionally, by measuring the electrolyte leakage we could demonstrate higher membrane damage in the PQ-stressed variants, coupled with various, but generally strong levels of rescue effect, caused by the SF pre-treatment (Figure 2). Considering this parameter, pepper shows the least damages, while tomato appears to be the most sensitive of the three species. The highest level of rescue is observed in Arabidopsis, and the lowest in pepper.

The maximum quantum yield and fluorescence decrease ratio were assessed by PAM fluorometry and showed considerable suppression of photosynthesis in PQ-only treated plants. Unlike the results from the other examination methods though, in this case pre-application of SF, despite producing a significant rescue effect, led to a weaker recovery of the observed parameters (Figure 4). Possible reasons could include the mode of action of PQ, which leads to superoxide production directly in the chloroplasts, and subsequently sensitizes the photosynthetic apparatus.

The combination of results from all physiological experiments lead to the conclusion that there is a common response to SF: when applied alone it does not seem to provoke any (or small) stimulating effects on the measured parameters in these particular conditions. This is probably due to the experimental setup, which includes a too short time window after the treatment, insufficient to allow monitoring of slower plant growth and development processes. Nevertheless, SF displays a universal protective effect against oxidative stress in all three species, which, however, has some specificities expressed in differences in the level of the rescue. The reason is probably due to the differences of the plants’ reactions to the stressor, combined with species-specific mechanisms via which SF achieves its shielding function.

For each of the studied species, the accumulation patterns of primary metabolites, visualized on unsupervised PCA plots, form four putative groups—one per each treatment (Supplementary Figure S2). Some of them exhibit partially overlapping areas, from which −SF/+PQ shows the most distinct separation. Using supervised sPLSDA graphs the focus falls on the specific differences between the groups and this creates distinct metabolic profiles for each treatment with much less interceding areas (Figure 5). A unique pattern is characteristic only for the −SF/+PQ variants, which reflects а massive response occurring after PQ-induction of oxidative stress. Remarkably, these PQ-provoked metabolic fingerprints between three different organisms are comparable. This is further supported by the separate inter-species grouping of the −SF/+PQ variants from all three organisms on the clustered heat maps: they are closer on the dendrogram than the intra-species treatment groups (Figure 7). Nevertheless, in some cases, several metabolites characteristically change in the −SF/+PQ variant of one species only (see Results). This might exemplify the differences in the modes of action, specific for every particular plant. The closer association of +SF/+PQ profiles to the control groups rather than to the stressed ones proves the high degree of rescue effect acquired by the SF pre-treatment, which prevents this variant from developing the distinct pattern of metabolic rearrangement, characteristic for ROS stressed plants. Assessment of sPLSDA component 1 values indicates that the metabolic changes induced by SF are strongly correlated with the evaluated plant phenotypes, an effect observed for all three plant species.

Considering the specific composition of the changed metabolomes and the metabolite categories within them, the major sub-group in the −SF/+PQ variant for Arabidopsis is formed by amino acids, all of which (11) are increasing. This is presumably due to protein degradation resulting from the oxidative stress-induced cell death [37]. For the +SF/+PQ variant, 10 of these AA also accumulate, but to a lesser amount, pointing to an incomplete rescue. The same observation is true for pepper, where two out of six selected metabolites are AAs, which are more elevated in −SF/+PQ and less in +SF/+PQ. Similarly, 10 out of the 11 PQ-modulated AAs in in tomato have higher abundance, with a well-pronounced recovery effect in the +SF/+PQ variant, where all AA amounts are tending to the base levels, with five of them slightly above and six—somewhat below the control quantities (Supplementary Figure S8).

Another significant metabolite group in Arabidopsis is formed by other organic acids and sugars, but this time most of them (8) decrease in −SF/+PQ and only five increase. For +SF/+PQ the numbers are almost the same: seven and six respectively. The rest of the biochemical substances are from the categories of polyols, amino alcohols, esters, nucleobases, etc., and one group (5) of unknown compounds.

Considering the selected tomato metabolites, they are distributed in the same general biochemical groups (AAs, organic acids, sugars, etc.), with a notable difference that only two compounds are down-accumulated in −SF/+PQ (aspartic acid and *cis*-3-caffeoyl-quinic acid), while nine decrease in +SF/+PQ: the abovementioned AAs, a keto acid (pyruvic acid), a sugar (rhamnose) and an unknown substance that is probably also a sugar.

The same biochemical groups, supplemented by one ketone (oxo-glutaric acid), are seen in pepper, but with two major differences—no unknown metabolites are present and all but the AAs increase in −SF/+PQ and slightly decrease in +SF/+PQ.

A notable common pattern is that salicylic acid (SA) considerably rises in the −SF/+PQ variant for all three species and goes back to normal in +SF/+PQ. SA is a phytohormone that plays a vital role in enhancement of the plant antioxidant defense system [38]. It can be regarded as а typical “stress metabolite”, with various functions in the regulation and mitigation of ROS induction, elicited by abiotic stresses such as heavy metals and UV-B [39,40,41,42], salinity, temperature, drought, etc. [38]. This explains its elevated levels in all PQ-treated samples.

It is noteworthy that four other differentially accumulated substances in Arabidopsis (beta alanine, gamma aminobutyric acid (GABA), *myo*-inositol and nicotinic acid) and three in tomato (beta-alanine, putrescine and trehalose) are considered stress metabolites as well, with various roles in oxidative and other types of stress. Beta-alanine is a well-documented stress response molecule involved in protecting plants from a variety of adverse conditions like extreme temperatures, drought, hypoxia, heavy metals, and even some biotic stresses [43]. GABA has а rapid stress-specific pattern of accumulation (in some cases with 300% for 15 s [44]) and plays a role in stress mitigation by triggering preventive metabolic changes, taking place before irreversible damage to tissues may occur [45]. During salt stress for example, it was demonstrated that the changes in GABA amounts precede the accumulation of other important salt-stress defense metabolites such as soluble sugars and proline [46]. *Myo*-inositol (and the precursor of trehalose - trehalose 6-phosphate) takes part in a broad regulatory network that contributes to oxidative stress homeostasis and plant stress tolerance [47]. Its broad range of derivatives can assume a dual role, acting both as structural lipids for the cell membrane and as diverse signal molecules, involved in a wide variety of cellular processes, including a crosstalk between sugar and lipid signaling. Trehalose itself is reported to play a role against oxidative stress caused by different abiotic stresses in several species, such as drought and salt in tomato [48] and heavy metal stress in tobacco [49]. Nicotinic acid and its metabolites are crucial for many vital functions as energy metabolism, oxidation-reduction reactions and various metabolic regulations. Moreover, nicotinic acid is the building block of many simple pyridine compounds [50]. Some of its metabolites take part in the signal transduction pathway regulating plants’ response to DNA strand breakage, which is caused mainly by oxidative stress [51]. Putrescine is known to reduce oxidative damage during strong abiotic stress, such as flooding [52], aluminium excess [53] and salt excess [54].

Overall, our study shows that SuperFifty’s protective role is not just limited to the model plant *Arabidopsis thaliana* but it successfully prevents PQ-induced oxidative damage in the important crops tomato and pepper as well. The metabolome reconfigurations, following different treatments, display patterns much more similar among the negative control, SF-treated and double-treated variants, than to the PQ-stressed one, suggesting that SF effectively mitigates the stress responses at the molecular level. These observations are equally valid for all three studied species, although some characteristic variations were observed.

Further steps need to be taken for additional elucidation of the exact mechanism through which SF positively affects the plants and mitigates oxidative stress. RNA-sequencing analyses, verified by qRT-PCR, could uncover more information about the transcriptomic changes, ruling the metabolomics rearrangements reported here. Furthermore, analyses of the activities of particular antioxidant enzymes and other stress markers could help narrow down the alternative pathways leading to the observed effects.

## 4. Materials and Methods

### 4.1. Plant Material, Growth Conditions and Treatments

*A. thaliana* Col-0 seeds were sown and germinated under plastic foil for 5 days. *C. annuum* cv. Kurtovska kapiya-1 and *S. lycopersicum* cv. Moneymaker seeds were treated with 10% solution of Na_3_PO_4_.12H_2_O by immersion with constant agitation for 120 min, after which they were rinsed twice with tap water and sown. The pots were kept under plastic foil until cotyledon emergence.

All plants were grown in pots, containing Rėkyva Remix Fine peat substrate and agro perlite in 2.5:1 ratio, supplemented with fertilizer mix comprising 1.2 g P_2_O_5_, 0.5 g K_2_SO_4_, 0.5 g NH_4_NO_3_ and 0.2 g MgSO_4_ per 1 L of potting mix. The pots with Arabidopsis were situated in a growth room under LED lights with 5000 K light temperature and 200 µmol/m^2^/s photosynthetic photon flux density (PPFD) with 16/8 (light/dark) photoperiod, temperature 22–23 °C and RH 70–85%. *Solanaceae* plants were grown under fluorescent lighting with 6500 K light temperature and 170 µmol/m^2^/s PPFD with 16/8 (light/dark) photoperiod, temperature 22–25 °C and RH 50–60%.

The experimental setup conditions were pre-defined with a series of preliminary experiments, determining the appropriate growth phase for treatment, scheme of applications and SF- (Supplementary Figure S1) and PQ- concentrations (data not shown).

After reaching the desired developmental stage (rosette stage for *A. thaliana* and fourth fully expanded leaf for *C. annuum* and *S. lycopersicum*), the plants were stimulated by foliar application as fine mist of 1% SuperFifty (BioAtlantis Ltd.) solution, supplemented with Tween 20 (Merck) in 0.05% concentration for *A. thaliana* and 0.1% for *Solanaceae*. SuperFifty is an extract of *Ascophyllum nodosum* (dry matter: 500 g/L, organic matter: 305 g/L minimum). After 24 h, oxidative stress was induced by foliar application of 15 µM and 25 µM (for *A. thaliana* and *Solanaceae*, respectively) methyl viologen (paraquat) dichloride hydrate (Sigma-Aldrich), supplemented with 0.05% Tween 20. This treatment was repeated on the following day. All control plants were treated with mock solution, containing only Tween 20.

The experiments were conducted in three independent repetitions, with each variant consisting of 24 biological replicates for *A. thaliana*, 16 for *C. annuum*, and 12 for *S. lycopersicum*.

### 4.2. Pulse-Amplitude-Modulation (PAM) Fluorometry

Maximum PSII quantum yield (QY_max_, F_v_/F_m_) was measured on full rosettes for Arabidopsis and abscised leaves for *Solanaceae*, with the help of FluorCam 800 MF - PSI (Photon Systems Instruments). Prior to measurements, the plants were dark adapted in a light-proof cupboard for 30 min. All consequent manipulations were performed under low-intensity diffused light, passed through green filters.

The stock Kautsky curve experiment with actinic light 1 source was used, with the following settings—Act1: 50% for Arabidopsis and Act1: 40% for *Solanaceae*. For all species, Shutter was set at 10 µs and Sensitivity at 20%. After the measurements were done, ROIs (Region of Interest) were manually specified using the selection tools in the FlourCam 7.0 software. Automatic background exclusion was performed before analysis.

### 4.3. Lesion Аrea Measurements

Damaged leaf area was quantified with the help of FIJI, a distribution of ImageJ [55]. The full leaf area and all lesions were defined as ROIs, after which the percentage of damaged area was determined.

### 4.4. Histochemical Detection of Hydrogen Peroxide by DAB Staining

Accumulation of H_2_O_2_ was detected using DAB (3, 3’-diaminobenzidine) staining solution, using 1 mg/mL DAB (Sigma-Aldrich) (adapted from [28] and [56]). The leaves were vacuum-infiltrated with the staining solution in a desiccator for 5 - 10 min, and then incubated at room temperature for 5 h. Following incubation, the DAB staining solution was replaced with absolute ethanol and the leaves were boiled at 100°C until chlorophyll was bleached. Leaves were rinsed with dH_2_O, and kept in 25% glycerol storage solution.

### 4.5. Electrolyte Leakage

Full rosettes for Arabidopsis and abscised leaves for *Solanaceae* were rinsed in dH_2_O, transferred to 50 mL tubes, containing 30 and 45 mL of dH_2_O, respectively, and incubated overnight at 4°C (Arabidopsis) and 15 min at room temperature on shaker (*Solanaceae*). Electric conductivity (EC) of the water was measured with HI 8733 (Hanna Instruments). All tubes were transferred to a water bath, and the samples were boiled for 15 min. Thereafter, EC was measured again. All measurements were made after the liquid temperature was equalized to RT, and the values were compensated with the conductivity of the used distilled water.

### 4.6. Gas Chromatography-Mass Spectrometry Analysis of Metabolites

Extraction and analysis by gas chromatography coupled with mass spectrometry was performed using the same equipment set up and protocol as described [57]. Briefly, frozen ground material was homogenized in 300 μL of methanol at 70 °C for 15 min and 200 μL of chloroform followed by 300 μL of water were added. The polar fraction was dried under vacuum, and the residue was derivatized for 120 min at 37 °C (in 40 μL of 20 mg/mL - 1 methoxyamine hydrochloride in pyridine) followed by a 30 min treatment at 37 °C with 70 μL of MSTFA. An auto-sampler Gerstel Multi-Purpose system (Gerstel GmbH & Co.KG) was used to inject the samples to a chromatograph coupled to a time-of-flight mass spectrometer (GC-MS) system (Leco Pegasus HT TOF-MS (LECO Corporation)). Helium was used as a carrier gas at a constant flow rate of 2 mL/s and gas chromatography was performed on a 30 m DB-35 column. The injection temperature was 230 °C and the transfer line and ion source were set to 250 °C. The initial temperature of the oven (85 °C) increased at a rate of 15°C/min up to a final temperature of 360 °C. After a solvent delay of 180 s, mass spectra were recorded at 20 scans s^−1^ with m/z 70–600 scanning range. Chromatograms and mass spectra were evaluated by using Chroma TOF 4.5 (Leco) and TagFinder 4.2 software. Metabolites were annotated based on a retention index calculation with deviation <5% and compared with the reference data of the Golm Metabolome Database, http://gmd.mpimp-golm.mpg.de [58].

### 4.7. Statistical and Data Analyses

All data were tabulated in Microsoft Excel 2016 (Microsoft), where the statistical processing was done. Any measurements falling outside the 1.5 interquartile range of their respective group were discarded as outliers. Figure 7, Figure S6 and Figure S7 were built with normalized to the negative control data sets. Data were expressed as mean ± standard error of the mean. Differences between two single treatments were tested by Welch’s t-test, with *p* < 0.05 considered statistically significant. Data from metabolomics analysis were normalized to the internal control (ribitol) and to their corresponding fresh weight. They were further analyzed and visualized using MetaboAnalyst [59], where normalization was set at “None” (default setting). All graphs, built with MetaboAnalyst were done using the default settings, with the fol-lowing exceptions: for creating the heat maps of Figure 7 and Supplementary Figure S6, distance measure “Pearson” and standardization “Autoscale samples” was used, together with the view option “Show only group averages”. For Supplementary Figure S7, the same settings plus view option “Use top: 25” was applied.

Comparison of sPLSDA values between treatment groups was performed using GraphPad Prism 6.0 (GraphPad Software) by means of one-way ANOVA followed by Tukey’s multiple comparisons test to adjust the *p*-values. Scatter plots of sPLSDA values and plant phenotypes were created using GraphPad Prism 6.0.

## 5. Conclusions

Pre-treatment of three different species with SF shows a clear protective effect from PQ-induced oxidative damage on several measured stress-related properties, such as: the number and surface of visibly damaged areas, H_2_O_2_ accumulation in the leaf tissues, plant photosynthetic parameters and cell-membrane integrity. The primary metabolite profiles of the stressed plants reveal both species-specific and common responses to the stressor, including accumulation of amino acids, most probably due to protein degradation, and elevation of stress metabolites like the phytohormone salicylic acid. On the other hand, the metabolome of SF-stimulated plants resembled the one of controls, regardless of PQ-application, which suggests that the biostimulant suppresses the molecular oxidative stress responses.

## Supplementary Materials

The following are available online at https://www.mdpi.com/2218-1989/11/1/24/s1, Figure S1: A single pre-treatment with increasing concentrations of SF protects *A. thaliana* rosette leaves from the damaging effect of the superoxide-inducing agent Paraquat, Figure S2: 2D PCA Scores plots for 89 metabolites of Arabidopsis, pepper and tomato, Figure S3: 3D sPLSDA Scores plots for 89 metabolites of Arabidopsis, tomato and pepper, Figure S4: Scatter plots of plant phenotype parameters versus sPLSDA component 2 values, Figure S5: Comparison of mean sPLSDA component 1 and component 2 values between treatment groups, Figure S6: Levels of primary metabolites, relative to the negative control, clustered for similarity of distribution patterns, Figure S7: Top 25 modulated metabolites (excerpt from Figure 7), Figure S8: Multiple selected metabolites by significance, Table S1: ANOVA, PC analysis for Figure 6 and Supplementary Figures S4 and S5, Table S2: Significances of all metabolites, each treatment against every other, calculated according Welch’s *t*-test.
