# Supplementary material for: An Ascophyllum nodosum-Derived Biostimulant Protects Model and Crop Plants from Oxidative Stress
Source: Metabolites. 2020 Dec 31;11(1):24. doi: 10.3390/metabo11010024 (PMC7824492; doi:10.3390/metabo11010024)
Supplement: Supplementary file 1 [file metabolites-11-00024-s001.zip › New folder/Captions.docx]

**Figure 1.** SuperFifty (SF) protects leaves from damage, caused by oxidative stress. Plants on the left are treated with paraquat (PQ), a herbicide that elevates the endogenous levels of ROS and triggers cell death (15 µM for Arabidopsis and 25 µM for tomato and pepper), and have clearly visible lesions. Plants on the right were pre-treated with 1% aqueous solution of SF, followed by the same treatment with PQ. Control plants, sprayed only with water (H_2_O) or only with 1% SF, show no signs of effect (pictures not shown).

**Figure 2.** Damage reduction, caused by SF pre-treatment. (**A**) SF reduces leaf lesion area in PQ-treated plants. The dead leaf surface, as a parameter indicating cell damage, of the four variants (as described in Materials and Methods) of Arabidopsis, pepper, and tomato (green, blue and yellow color bars, respectively) was assessed with the ImageJ software. n=12 for Arabidopsis, n=48 for pepper and tomato. (**B**) SF prevents excess ion leakage in PQ-treated plants. Measurement of electrolyte leakage in leaves of Arabidopsis, pepper and tomato exposed to four different treatments, relative to control. n=36 for Arabidopsis, n=48 for pepper and tomato. Data are means ±SEM (standard error of the mean) of three biological replicates. Statistical significance relative to controls from the same species is indicated with asterisks as follows: P ≤ 0.0001 (****), P ≤ 0.001 (***), P ≤ 0.01 (**), P ≤ 0.05 (*).

**Figure 3.** SF protects from accumulation of H_2_O_2_ in PQ treated plants. (**A**) DAB-stained leaves of Arabidopsis, pepper and tomato exposed to different treatments (**B**) The percentage of DAB stained area of the four variants of Arabidopsis, tomato, and pepper was assessed with the ImageJ software as described in Materials and Methods (n=6). Data are means ±SEM of three biological replicates. Statistical significance relative to controls from the same species is indicated with asterisks as follows: P ≤ 0.0001 (****), P ≤ 0.001 (***), P ≤ 0.01 (**), P ≤ 0.05 (*).

**Figure 4.** SF pretreatment alleviates the reduction of chlorophyll fluorescence parameters induced by oxidative stress. The maximum quantum yield (**A**) and fluorescence decrease ratio (**B**) in dark-adapted Arabidopsis, pepper and tomato plants (n=12), treated or not with SF and PQ, were measured by PAM fluorometry. Data are means ±SEM of three biological replicates. Statistical significance relative to controls from the same species is indicated in the Table as follows: P ≤ 0.0001 (****), P ≤ 0.001 (***), P ≤ 0.01 (**), P ≤ 0.05 (*).

**Figure 5.** 2D sPLSDA Scores plots for 89 metabolites of Arabidopsis, pepper and tomato. The -SF/+PQ treatment (light green horizontally arranged clusters) always groups separately from the others, which show much more similar distribution and partial or complete overlapping (all others, generally vertical clusters) (n=6). AT – *Arabidopsis thaliana*, CA – *Capsicum annuum*, SL – *Solanum lycopersicum*.

**Figure 6:** Scatter plots of plant phenotype parameters versus sPLSDA component 1 values. Mean sPLSDA component 1 values were compared between treatment groups using one-way ANOVA, with Tukey’s multiple comparisons test applied to correct for multiple testing. For all three plant species, a significant difference was observed between the -SF/+PQ (oxidatively stressed) and all other treatment groups. This was observed for both the sPLSDA component 1 values and for all plant phenotypes measured. Horizontal error bars denote the standard error of the mean (SEM) of sPLSDA component 1 values. Vertical error bars denote the SEM of phenotype parameters. Asterisks denote statistically significant differences between treatment groups and the untreated control (-SF/-PQ) as follows: P ≤ 0.0001 (****), P ≤ 0.001 (***), P ≤ 0.01 (**), P ≤ 0.05 (*). Horizontal and vertical asterisks denote significant differences between treatment groups and the untreated control for phenotype parameters and sPLSDA component 1 values respectively. Full data are given in Supplementary Materials (Supplementary Figures S4 and S5) and Supplementary Table S1.

**Figure 7.** Levels of primary metabolites, relative to the negative control (-SF/-PQ) for each of the species, clustered for similarity of distribution patterns. The length of the lateral arms of the dendrograms represent the degree of resemblance. Significance can be seen in Supplementary Table S2 (n=6). AT – *Arabidopsis thaliana*, CA – *Capsicum annuum*, SL – *Solanum lycopersicum*.

**Supplementary** **Figure S1.** A single pre-treatment with increasing concentrations of SF protects *A. thaliana* rosette leaves from the damaging effect of the superoxide-inducing agent Paraquat. (**A**) Phenotype: Plants, pre-treated with increasing concentrations of SF, followed by PQ treatment. (Representative examples from three independent experiments shown). (**B**) PAM fluorimetry: Maximum Quantum Yield and Fluorescence Decline Ratio were determined *in-situ*. Lower values indicate higher degree of stress. (**C**) Mass of rosette leaves: Mass increases with application of higher concentrations of SF, reaching a plateau after 0.2% at about 87-89% of mock-treated. (**D**) Electrolyte leakage: EL was determined on detached whole rosettes. Higher values correspond with higher degree of damaged and dead cells. Data are means ±SEM of three biological replicates. Significance (Welch’s T-test) is calculated against the corresponding mock-treated control for each SF-concentration and is indicated as follows: P ≤ 0.0001 (****), P ≤ 0.001 (***), P ≤ 0.01 (**), P ≤ 0.05 (*).

**Supplementary** **Figure S2.** 2D PCA Scores plots for 89 metabolites of Arabidopsis, pepper and tomato. The -SF/+PQ treatment (light green clusters) groups mostly aside from the other clusters, with the exception of pepper, where -SF/+PQ overlaps all groups due to its high variability. The rest of the treatments display higher resemblances and hence - partial or complete overlapping (n=6). AT – *Arabidopsis thaliana*, CA – *Capsicum annuum*, SL – *Solanum lycopersicum*.

**Supplementary** **Figure S3.** 3D sPLSDA Scores plots for 89 metabolites of Arabidopsis, tomato and pepper. A better separate clustering of each treatment group in the three-dimensional space can be observed (add-on for Fig. 5). AT – *Arabidopsis thaliana*, CA – *Capsicum annuum*, SL – *Solanum lycopersicum*.

**Supplementary** **Figure S4.** Scatter plots of plant phenotype parameters versus sPLSDA component 2 values. Mean sPLSDA component 2 values were compared between treatment groups using one-way ANOVA, with Tukey’s multiple comparisons test applied to correct for multiple testing. Horizontal error bars denote the standard error of the mean (SEM) of sPLSDA component 2 values. Vertical error bars denote the SEM of phenotype parameters. Asterisks denote statistically significant differences between treatment groups and the untreated control (-SF/-PQ) as follows: P ≤ 0.0001 (****), P ≤ 0.001 (***), P ≤ 0.01 (**), P ≤ 0.05 (*). Horizontal and vertical asterisks denote significant differences between treatment groups and the untreated control for phenotype parameters and sPLSDA component 2 values respectively. Full data are given in Supplementary Table S1.

**Supplementary** **Figure S5.** Comparison of mean sPLSDA component 1 and component 2 values between treatment groups. sPLSDA component 1 and component 2 values are plotted for the metabolome datasets. Plotted are six biological replicates per treatment. Mean component 1 and component 2 values were compared between groups by means of one-way ANOVA followed by Tukey's multiple comparisons test. An absence of letter sharing between the groups denotes a statistically significant difference (P≤0.05) between those groups. Error bars denote standard error of the mean (SEM) of component values. Full data are given in Supplementary Table S1.

**Supplementary** **Figure S6.** Levels of primary metabolites, relative to the negative control, clustered for similarity of distribution patterns. The length of the lateral arms of the dendrograms represent the degree of resemblance. Significance can be seen in Supplementary Table S2 (n=6).

**Supplementary** **Figure S7.** Top 25 modulated metabolites (excerpt from Fig. 7). The metabolites with the most different distribution patterns in –SF/+PQ across all species are framed in black. AT – *Arabidopsis thaliana*, CA – *Capsicum annuum*, SL – *Solanum lycopersicum*.

**Supplementary** **Figure S8.** Multiple selected metabolites by significance: -SF/+PQ significant vs. -SF/-PQ (stress-dependent response), +SF/-PQ not significant vs. -SF/-PQ (not influenced by SF alone), +SF/+PQ significant vs. -SF/+PQ (rescue effect of the SF-pretreatment), for each of the species.
