# Supplementary figures and images for: An Ascophyllum nodosum-Derived Biostimulant Protects Model and Crop Plants from Oxidative Stress
Source: Metabolites. 2020 Dec 31;11(1):24. doi: 10.3390/metabo11010024 (PMC7824492; doi:10.3390/metabo11010024)

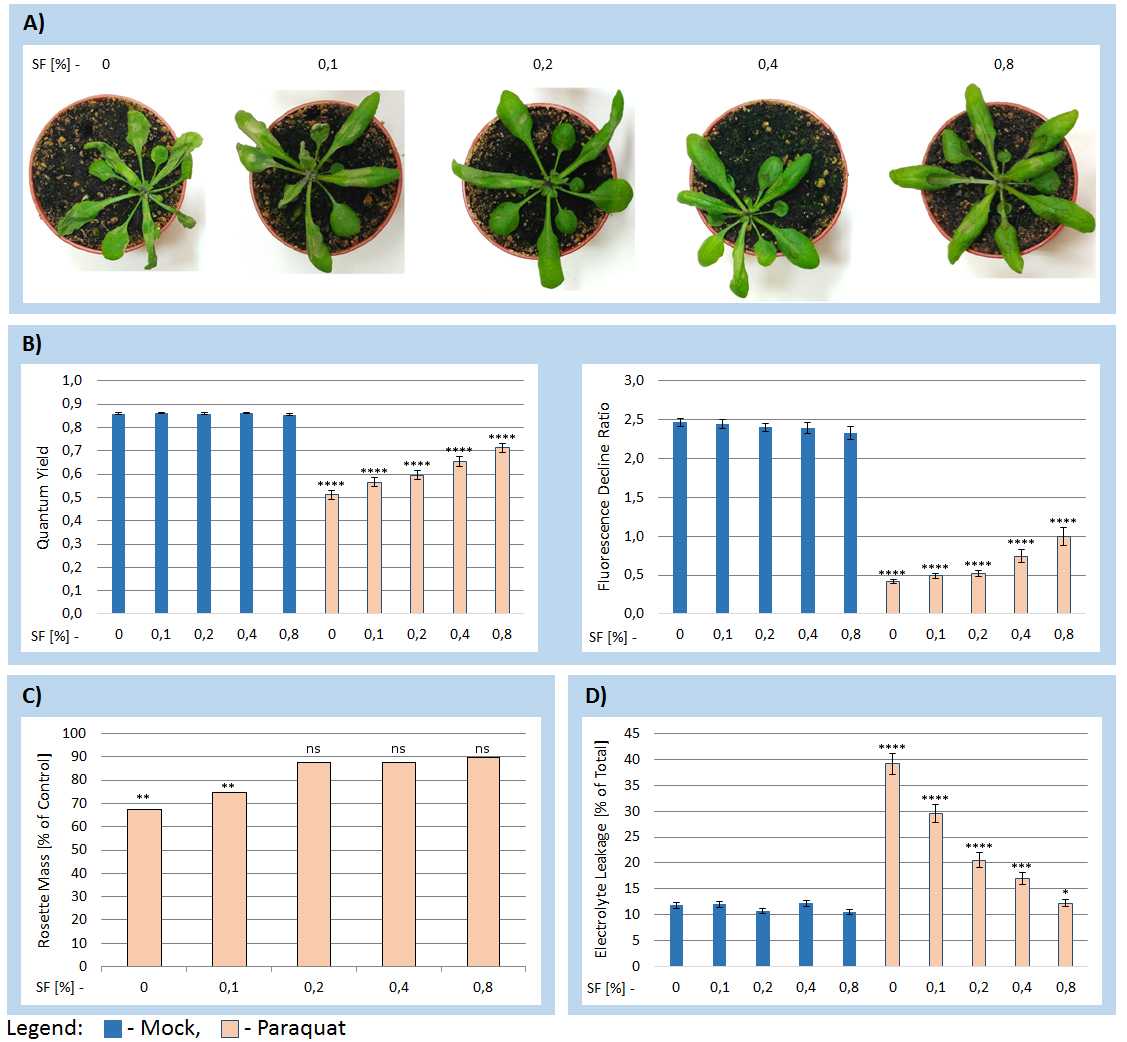

Supplement: Supplementary file 1 [file metabolites-11-00024-s001.zip › New folder/Supplementary_S1.jpg]

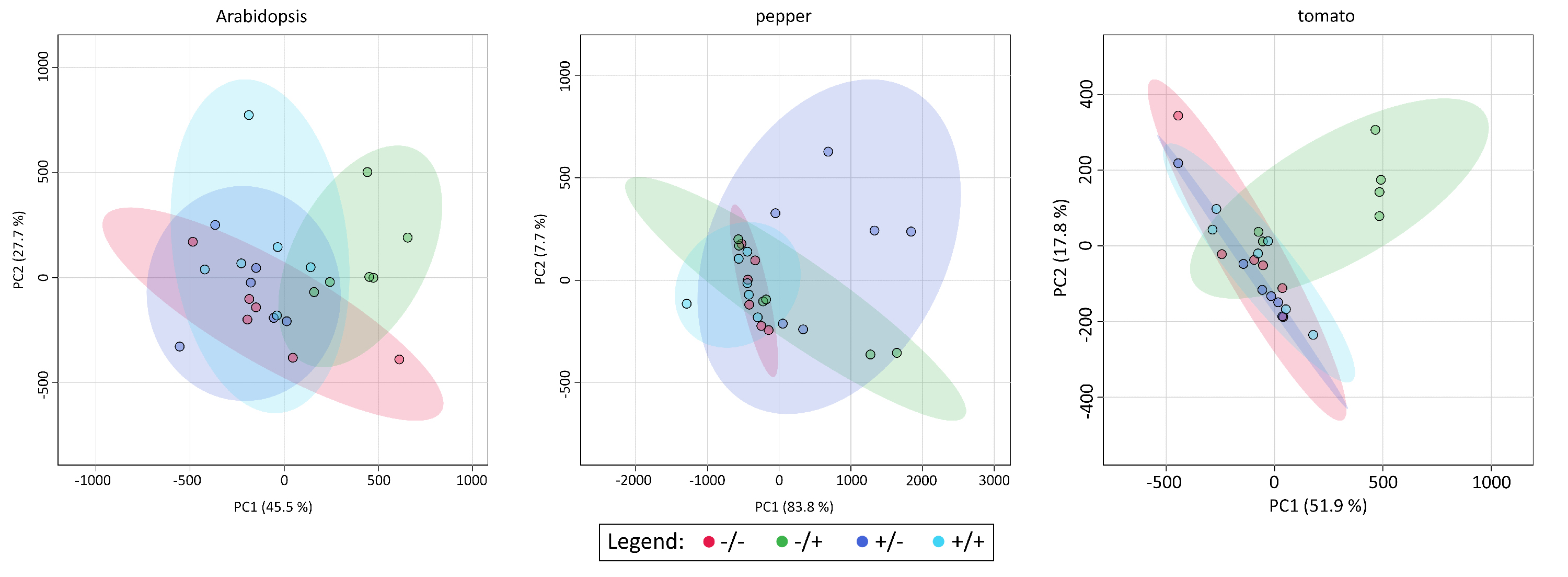

Supplement: Supplementary file 1 [file metabolites-11-00024-s001.zip › New folder/Supplementary_S2.jpg]

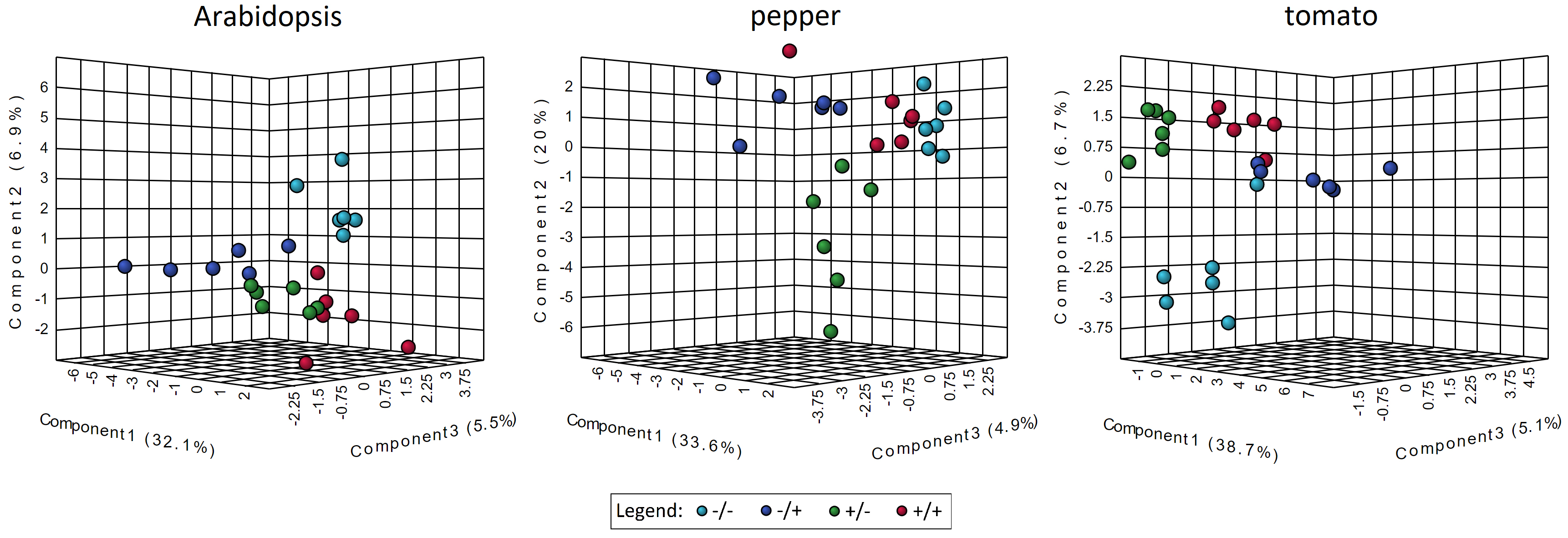

Supplement: Supplementary file 1 [file metabolites-11-00024-s001.zip › New folder/Supplementary_S3.jpg]

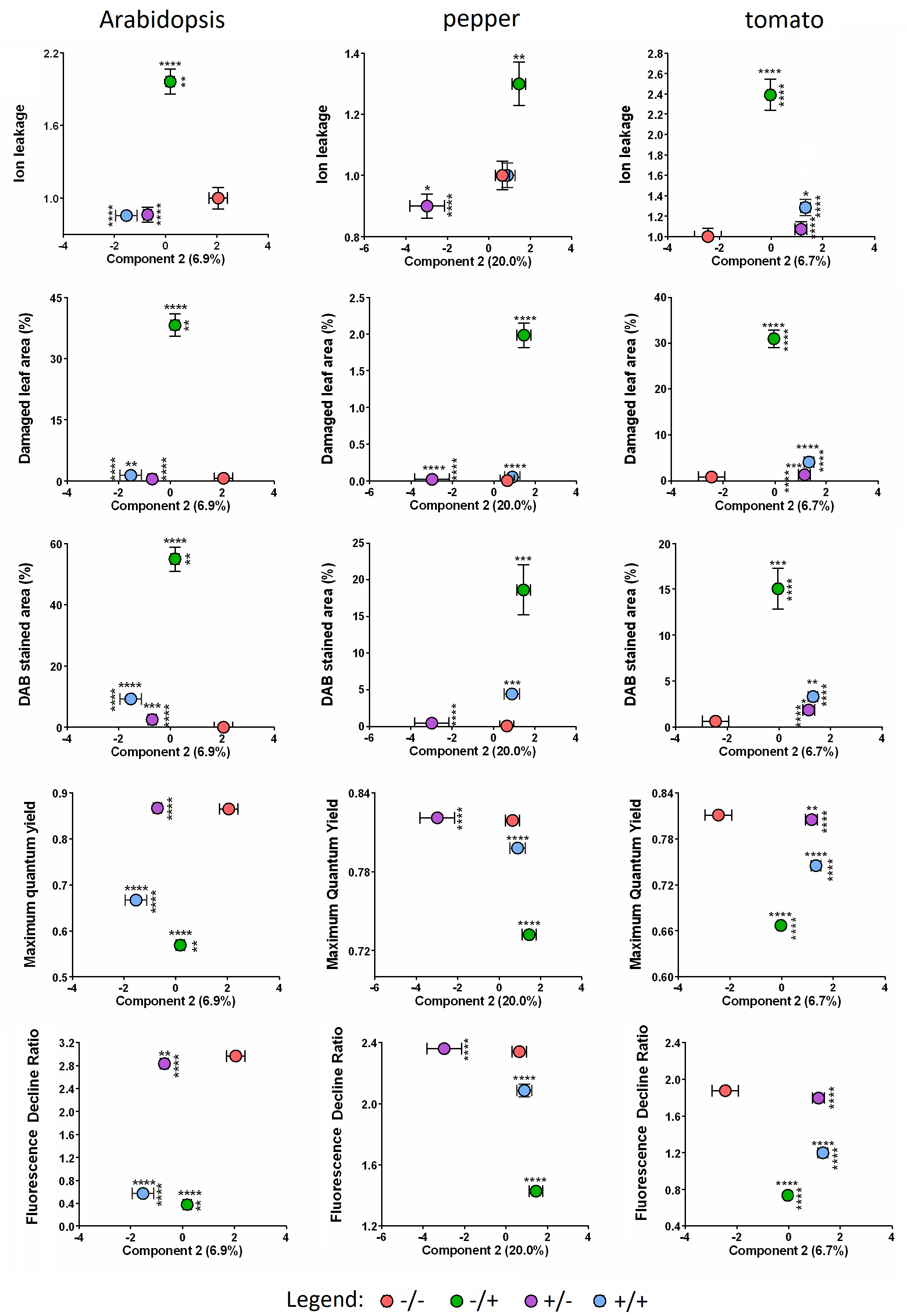

Supplement: Supplementary file 1 [file metabolites-11-00024-s001.zip › New folder/Supplementary_S4.jpg]

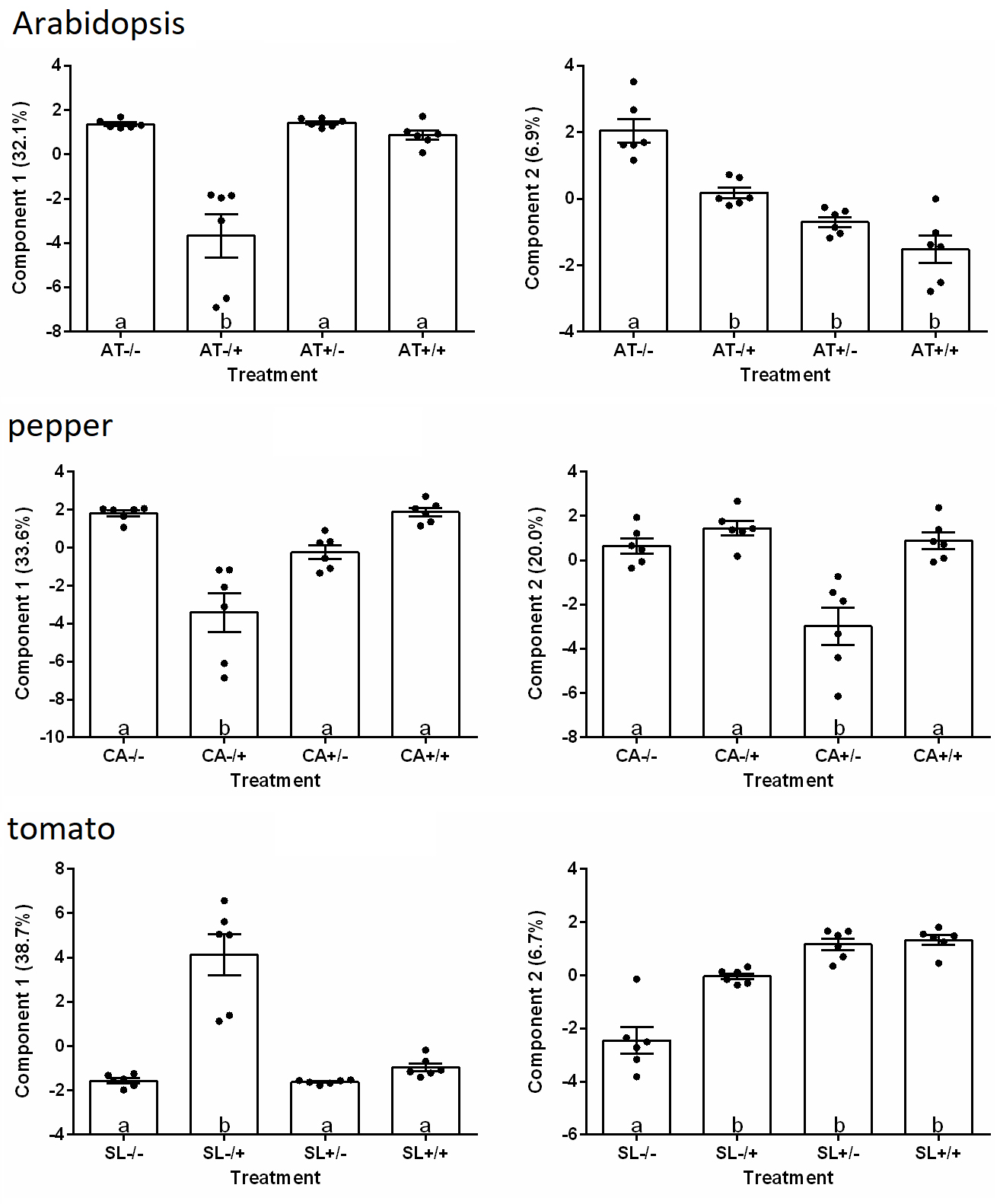

Supplement: Supplementary file 1 [file metabolites-11-00024-s001.zip › New folder/Supplementary_S5.jpg]

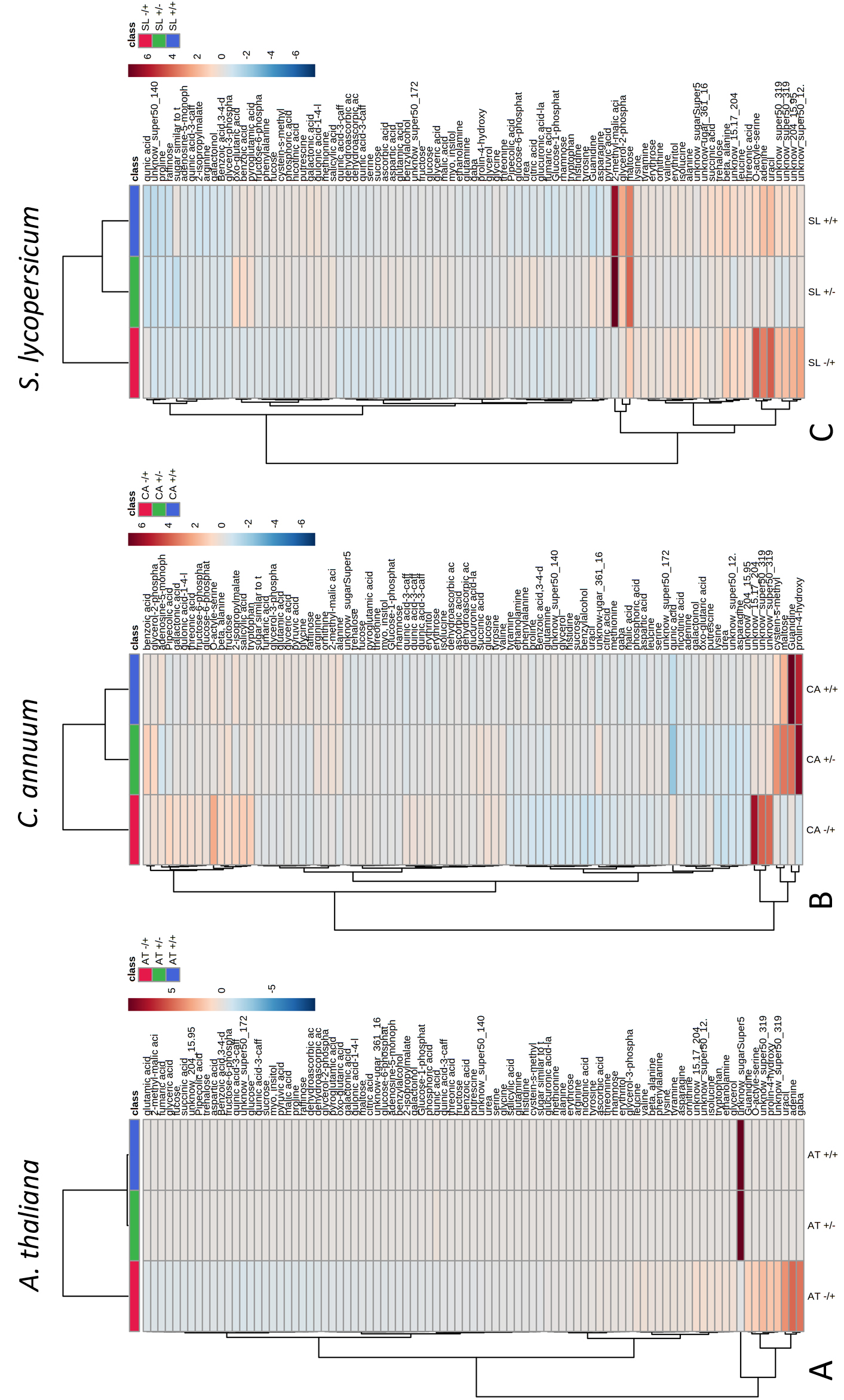

Supplement: Supplementary file 1 [file metabolites-11-00024-s001.zip › New folder/Supplementary_S6.jpg]

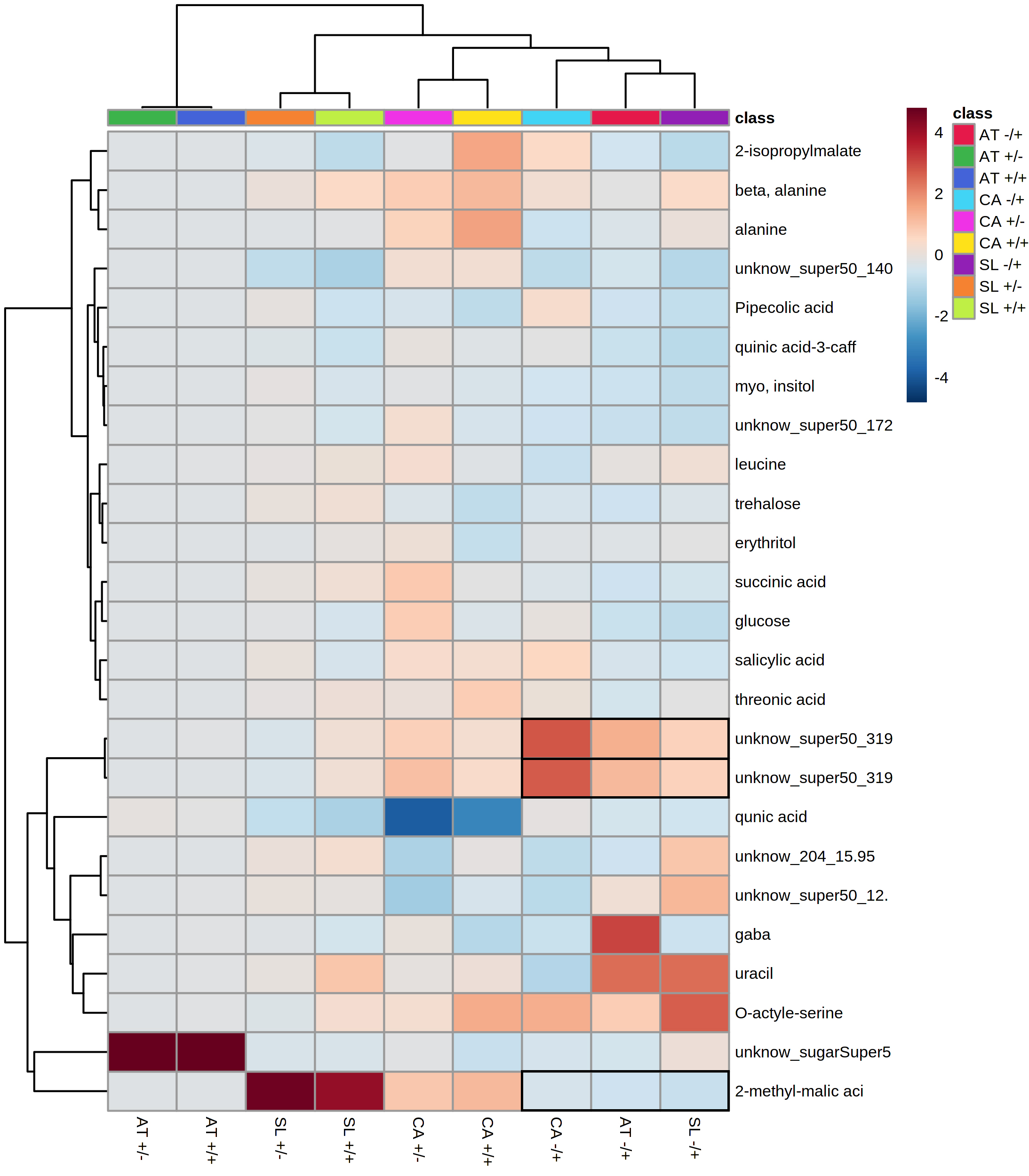

Supplement: Supplementary file 1 [file metabolites-11-00024-s001.zip › New folder/Supplementary_S7.jpg]

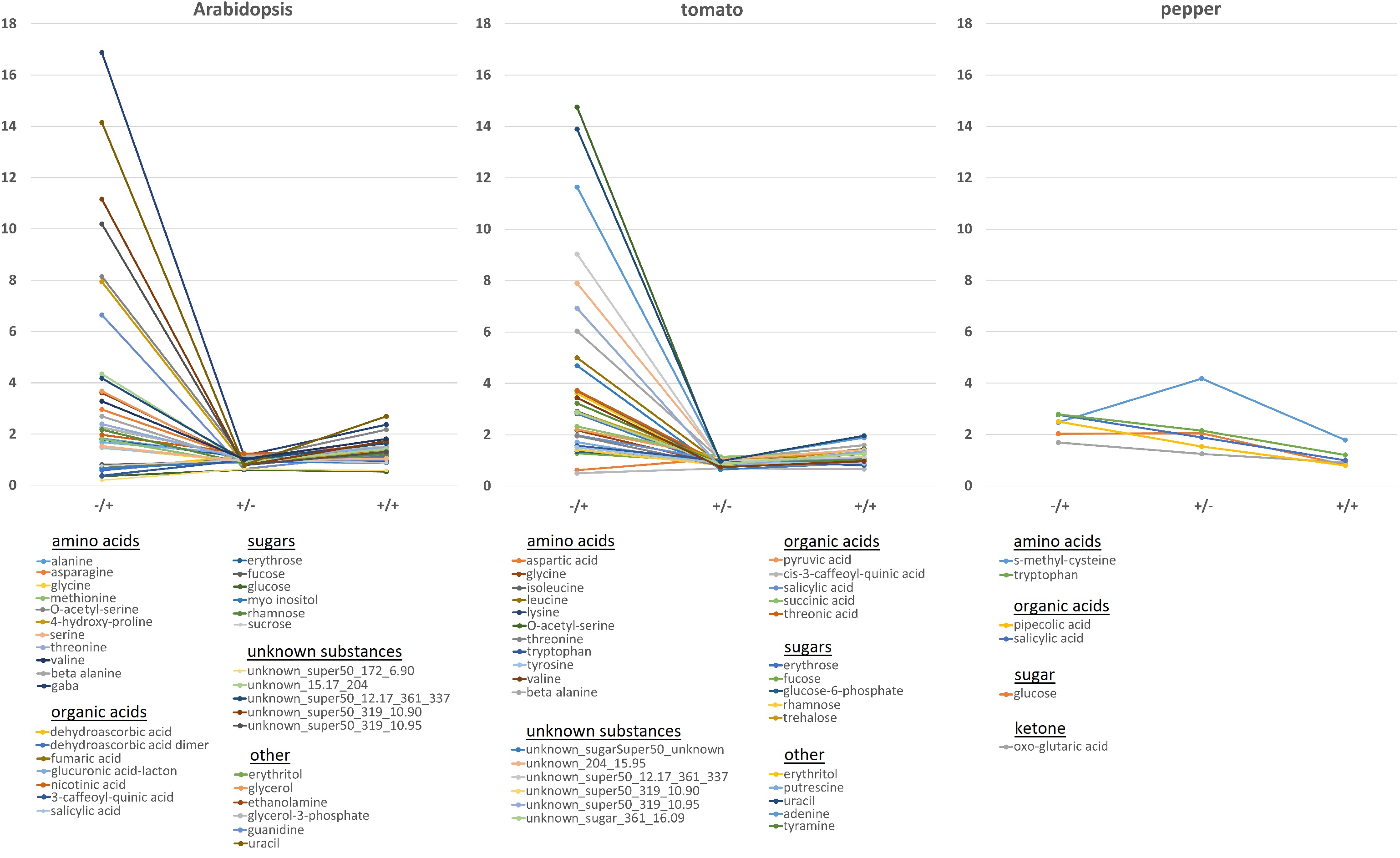

Supplement: Supplementary file 1 [file metabolites-11-00024-s001.zip › New folder/Supplementary_S8.jpg]
